# Assessment and predictors of physical functioning post-hospital discharge in survivors of critical illness

**DOI:** 10.1186/s13613-016-0187-8

**Published:** 2016-09-20

**Authors:** Kevin J. Solverson, Christopher Grant, Christopher J. Doig

**Affiliations:** 1 Department of Critical Care Medicine, Cumming School of Medicine, University of Calgary, 3134 Hospital Drive NW, Calgary, AB T2N 2T9 Canada; 2 Division of Physical Medicine and Rehabilitation, Cumming School of Medicine, University of Calgary, 3134 Hospital Drive NW, Calgary, AB T2N 2T9 Canada; 3 Department of Community Health Sciences, Cumming School of Medicine, University of Calgary, 3134 Hospital Drive NW, Calgary, AB T2N 2T9 Canada

**Keywords:** Critical care, Muscle weakness, Muscle strength dynamometer, Sepsis, Recovery of function, Adult

## Abstract

**Background:**

Prior studies of physical functioning after critical illness have been mostly limited to survivors of acute respiratory distress syndrome. The purpose of this study was to objectively assess muscle strength and physical functioning in survivors of critical illness from a general ICU and the associations of these measures to health-related quality of life (HRQL), mental health and critical illness variables.

**Methods:**

This was a prospective cohort study of 56 patients admitted to a medical ICU (length of stay ≥4 days) from April 1, 2009, and March 31, 2010. Patients were assessed in clinic at 3 months post-hospital discharge. Muscle strength and physical functioning were measured using hand-held dynamometry and the 6-min walk test. HRQL was assessed using the short-form 36 (SF-36) and EuroQol-5D (EQ-5D) questionnaires.

**Results:**

Three months post-hospital discharge, median age- and sex-matched muscle strength was reduced across all muscle groups. The median 6-min walk distance was 72 % of predicted. Physical functioning was associated with reductions in self-reported HRQL (SF-36, EQ-5D) and increased anxiety. Univariate regression modeling showed that reduced muscle strength and 6-min walk distance were associated with sepsis but not ICU length of stay. Multivariate regression modeling showed that sepsis and corticosteroid use were associated with a reduced 6-min walk distance, but again ICU length of stay was not.

**Conclusions:**

Survivors of critical illness have reduced strength in multiple muscle groups and impaired exercise tolerance impacting both HRQL and mental health. These outcomes were worsened by sepsis and corticosteroid use in the ICU but not ICU length of stay. Interventions to minimizing the burden of sepsis in critically ill patients may improve long-term outcomes.

## Background

As more patients are surviving critical illness, examining longer-term outcomes becomes increasingly important. There is increasing evidence that critical illness survivors have impaired physical functioning, increased prevalence of mental health disorders and reduced health-related quality of life (HRQL) [1–6].

Critically ill patients have been shown to develop multifactorial weakness termed ICU-acquired weakness (ICUAW), and these patients are at risk of prolonged ICU lengths of stay, increased mechanical ventilation time, prolonged weakness and poor hospital outcomes [7–13]. Muscle biopsies taken during critical illness have shown wasting of the muscle fibers and increased catabolic metabolism [14–17]. Risk factors for ICUAW include prolonged immobility, mechanical ventilation, corticosteroid and neuromuscular blockade administration and cytokine-related injury from systemic inflammation [5, 7, 9, 10, 17–24]. However, the duration of physical impairment after hospital discharge secondary to critical illness and the predictors of severity remain unclear.

Studies that have objectively examined long-term physical function have primarily focused on survivors of acute respiratory distress syndrome (ARDS) and not the general ICU survivor population. Long-term physical function of ARDS survivors was first objectively described by Herridge et al. [5] landmark paper using the 6-min walk test (6MWT). Recent post-ICU studies have used manual muscle testing (MMT) for the assessment of muscle strength in addition to the 6MWT; however, MMT has limitations including a ceiling effect in less severe muscle weakness [25, 26]. Isokinetic muscle strength testing using hand-held dynamometry has been shown to be a more sensitive and objective method of strength testing compared to MMT [25, 27–30]. However, to date hand-held dynamometry has not been used in the ICU survivor population, despite its common use in other patient populations [31–34].

The goal of our study was to objectively examine muscle strength and physical functioning using hand-held dynamometry and the 6MWT in critical illness survivors 3 months after hospital discharge. Additionally, we sought to determine whether muscle strength or physical functioning was associated with HRQL, mental health or critical illness variables such as severity and type of illness and ICU length of stay.

## Methods

### Design

This was a prospective longitudinal cohort study of patients who were admitted to a 25-bed multidisciplinary tertiary referral ICU, which also served as the trauma center for southern Alberta [35]. Enrollment occurred between April 1, 2009, and March 31, 2010. At the time of study enrollment, there were two full-time equivalent physiotherapists; however, no patients were mobilized while intubated in the ICU. The initiation of physiotherapy was up to the discretion of the attending physician.

Patients assessed in the ICU follow-up clinic were adult patients (≥18 years), admitted to the ICU with a minimum 4-day length of ICU stay. Patients were excluded if they had traumatic brain injuries, spinal cord injuries, pre-existing neurocognitive or neuromuscular disorders, acute strokes or lived outside of the immediate municipality of Calgary. Patients in the ICU were screened for eligibility during the initial 48 h and approached for follow-up once they had been admitted to the ICU for a minimum of 4 days. Due to limited capacity in the ICU follow-up clinic, patients were enrolled consecutively until clinical capacity was met at which point screening would be temporally suspended. Patients enrolled in the ICU were assessed at 3 months after hospital discharge. A total of 61 patients met inclusion criteria, 4 patients declined follow-up, and 1 patient was lost to follow-up. Attendance at the clinic was presented as a natural continuation of care following hospitalization, which individuals had the option to refuse. We sought permission from these patients to include their clinical data in our study. This study was approved by the University of Calgary Human Research Ethics Board (ID# E-22574), and written informed consent was obtained from all patients.

### Instruments/questionnaires

A single trained individual (author KS) assessed peripheral muscle strength using hand-held dynamometry. The JAMAR (5030J1) hydraulic hand dynamometer and the Chatillon dynamometer (K-MSC-200) were used for all muscle strength assessments. All measurements were taken in kilograms to the nearest hundredth of a kilogram. Each muscle group (handgrip, triceps, biceps, ankle dorsiflexors, hamstrings and quadriceps) was measured according to previously validated protocols [36]. For each muscle group, patients were asked to exert a maximal effort for 3–4 s against the dynamometer, which was in a fixed position. Three measurements were collected for each muscle group, alternating between patient sides between measurements. For each muscle group, the highest force generated on the patient’s dominant side was used for analysis. The National Isometric Muscle Strength Database Consortium [36] was used for age- and sex-standardized normative values. The 6MWT was used to assess overall physical functioning. Previously published guidelines and procedures were followed [37], and age and sex normative values were obtained from Enright et al. [38].

The assessment of HRQL was done using short-form 36 (SF-36) and EuroQol-5D (EQ-5D) surveys [39]. The SF-36 survey was reported as each domain ranging from 0 to 100 (higher scores indicate better HRQL), and the physical and mental composite scores (PCS, MCS) were standardized to Canadian population norms (score of 50 represents the average normative value) [40]. The Hospital Anxiety and Depression Scale (HADS) was used to assess anxiety and depression, scores ranging from 0 to 21 [41]. A score >7 on either the anxiety or depression section indicates severe symptoms. All questionnaires were administered at the time of the patient’s clinical visit.

### Statistical analysis

All clinical data were entered into a study-specific database. These data were merged with clinical and outcome data from an ICU-specific longitudinal database, details described elsewhere [42]. All data analysis was performed using Stata 11.0 (Stata Corp, College Station, TX). Descriptive statistics were used to report patient demographics, hand-held dynamometry testing and the 6MWT.

Multiple linear regression models were used to assess the association between the strength of each muscle group (independent variable), 6MWT, EQ-5D and the SF-36 (dependent variables). The EQ-5D domains were modeled as a dichotomous variable (0 = no problems, 1 = reporting any problems). Multiple univariate and a single multivariate linear regression analysis assessed the association of the following ICU risk factors (dependent variables) to patient’s predicted peripheral muscle strength or 6MWT (independent variables): (1) ICU length of stay (LOS), (2) hospital LOS, (3) severity of illness as measured by the mean Acute Physiology and Chronic Health Evaluation (APACHE) II score, (4) degree of organ failure measure by the Sequential Organ Failure Assessment (SOFA) score, (5) presence of sepsis (defined using the 2001 American College of Chest Physicians guideline [43]), (6) presence of ARDS (defined as a PaO2:FiO2 ratio of 200 or less while mechanically ventilated with evidence of airspace disease in all four quadrants on chest radiograph), (7) any corticosteroid use, (8) any neuromuscular blocker use (9), duration of ventilation and (10) Functional Comorbidity Index score. For the multivariate linear regression, all ICU risk factors were analyzed in the model and variables that had a *p* value <0.05 were carried forward in the analysis to create the final model.

## Results

During the study period, 56 patients were seen in the ICU follow-up clinic. The median (IQR) age was 61 years (41, 68), and 54 % were males (Table 1). Prior to ICU admission, 57 % of patients had one or more pre-existing comorbidity and 100 % were living independently at home. The median (IQR) first APACHE II score and maximum SOFA scores were 19 (16, 24) and 11 (9, 14), respectively. Only 18 % of patients received corticosteroids, and 21 % received neuromuscular blockers anytime while in the ICU. No patient with ARDS received corticosteroids. The median (IQR) days of mechanical ventilation and ICU and hospital LOS were 8 days (4, 11), 11 days (6, 15) and 12 days (8, 12), respectively. The median (IQR) time to follow-up was 72 days (54, 92) after hospital discharge.

**Table 1:** Baseline patient characteristics

Variable	All patients (n = 56)^a^
*Baseline characteristics prior to admission*	
Age (year)	61 (41, 68)
Male [n (%)]	30 (54)
Pre-existing comorbidity [n (%)]	32 (57)
Charlson comorbidity index score	3 (1, 4)
Functional Comorbidity Index score	1 (0, 2)
Living independently at home [n (%)]	56 (100)
Body mass index (kg/m^2^)	26 (22, 33)
*ICU characteristics*	
Diagnosis at ICU admission [n (%)]	
Respiratory failure	37 (66)
Intra-abdominal infection	7 (13)
Urologic infection	3 (5)
Poly-trauma	4 (7)
Other	5 (9)
Sepsis present during admission [n (%)]	39 (70)
ARDS present during admission [n (%)]	13 (23 %)
Any corticosteroid use [n (%)]	10 (18)
Daily dose corticosteroids if received any, mg (prednisone equivalent)	57 (35, 82)
Any neuromuscular blocker use [n (%)]	12 (21)
APACHE II score	19 (16, 24)
SOFA score maximum value	11 (9, 14)
Length of mechanical ventilation (day)	8 (4, 11)
Length of ICU stay (day)	11 (6, 15)
Length of hospital stay (day)	12 (8, 21)
Time to follow-up after hospital discharge (day)	72 (54, 92)

*ARDS* acute respiratory distress syndrome, *APACHE II* Acute Physiology and Chronic Health Evaluation, *SOFA* Sequential Organ Failure Assessment, *ICU* intensive care unit

^a^Reported as median (interquartile range) unless specified

Patient’s median dynamometry-measured muscle strength was reduced across all muscle groups when compared to age- and sex-matched data (Table 2). The median (IQR) percent-predicted strength of the handgrips 61 % (25, 108), quadriceps 59 % (35, 88) and the ankle dorsiflexors 62 % (42, 92) all showed the greatest impairment 3 months after critical illness. For all the muscle groups, over 50 % of the patients did not achieve 80 % of their age- and sex-matched predicted strength. The median (IQR) distance walked in 6 min and percent predicted were 382 m (291, 480) and 70 % (53, 88), respectively. In total, 55 % of patients did not achieve at least 80 % of their age- and sex-matched predicted 6MWT distance. The maximum strength results of each muscle group statistically correlated with the 6MWT distance.

**Table 2:** Muscle strength and physical functioning of critical illness survivors at 3 months after hospital discharge measured using hand-held dynamometry and the 6-min walk test

Variable (n = 56)	Measurement (median, IQR)	% predicted (median, IQR)	% of patients < 80% predicted strength	Correlation with the 6MWT (R^2^, p value)
Maximum muscle strength (kg)^a^				
Grip	20.4 (9.1, 30.6)	61 % (25, 108)	60	0.28 (<0.001)
Triceps	12.3 (8.7, 16.7)	71 % (45, 126)	52	0.31 (<0.001)
Biceps	14.5 (11.7, 19.0)	72 % (45, 126)	52	0.28 (<0.001)
Hamstrings	13.2 (10.3, 17.6)	77 % (47, 114)	53	0.30 (<0.001)
Quadriceps	20.3 (16.4, 25.6)	59 % (35, 88)	69	0.14 (0.013)
Ankle dorsiflexors	13.7 (10.6, 17.2)	62 % (42, 92)	57	0.13 (<0.001)

*6MWT* 6-min walk test

^a^Strength results reported as the maximum force generated on the dominant body side

Table 3 outlines individual regression analyses evaluating the associations of physical functioning to HRQL and mental health. Reporting problems in the domains mobility (p = 0.003), usual activities (p = 0.006), pain and discomfort (p = 0.031) of the EQ-5D were each associated with reduced predicted 6MWT distance. Patient’s overall impression of their global health using the EQ-5D VAS was also associated with performance on the 6MWT (p = 0.025). Among the muscle groups strength tested, grip (p = 0.030), hamstring (p = 0.021) and quadriceps (p = 0.019) weakness were associated with increasing impairment on the mobility domain of the EQ-5D.

**Table 3:** Associations of physical functioning and health-related quality of life at 3 months after hospital discharge

Variable n = 50^a^	6-Minute walk test	Grip	Triceps	Biceps	Hamstrings	Quadriceps	Ankle dorsiflexors
EuroQol-5D^b^							
Mobility	−0.211 (0.003)	−0.297 (0.030)	−0.223 (0.144)	−0.228 (0.097)	−0.283 (0.021)	−0.252 (0.019)	−0.140 (0.125)
Self-care	−0.177 (0.067)	−0.132 (0.435)	−0.170 (0.372)	0.165 (0.336)	−0.198 (0.190)	−0.177 (0.197)	−0.142 (0.212)
Usual activities	−0.177 (0.006)	−0.166 (0.153)	−0.044 (0.736)	−0.061 (0.606)	−0.103 (0.335)	−0.070 (0.446)	−0.127 (0.105)
Pain/discomfort	−0.125 (0.031)	−0.020 (0.855)	−0.011 (0.929)	−0.105 (0.345)	−0.096 (0.359)	−0.072 (0.412)	−0.031 (0.688)
Anxiety/depression	−0.123 (0.079)	−0.099 (0.476)	−0.120 (0.421)	−0.065 (0.630)	−0.021 (0.866)	−0.001 (0.994)	−0.007 (0.941)
Visual analog scale^c^	0.050 (0.025)	0.324 (0.428)	0.028 (0.534)	0.018 (0.662)	0.026 (0.480)	−0.010 (0.759)	−0.009 (0.746)
Short-from 36							
Physical functioning^d^	0.005 (0.001)	0.006 (0.019)	0.004 (0.178)	0.004 (0.112)	0.005 (0.026)	0.004 (0.062)	0.003 (0.063)
Physical composite score^e^	0.010 (0.001)	0.008 (0.185)	0.000 (0.997)	0.007 (0.266)	0.009 (0.105)	0.008 (0.113)	0.004 (0.320)
Mental composite score^e^	0.005 (0.118)	0.010 (0.128)	0.003 (0.712)	0.006 (0.409)	0.005 (0.416)	0.005 (0.308)	0.004 (0.453)
HADS^f^							
Anxiety	−0.029 (0.001)	−0.023 (0.176)	−0.025 (0.179)	−0.025 (0.130)	−0.023 (0.123)	−0.018 (0.16)	−0.014 (0.251)
Depression	−0.016 (0.098)	−0.033 (0.080)	−0.008 (0.718)	−0.023 (0.214)	−0.012 (0.174)	−0.027 (0.067)	0.010 (0.450)

*ICU* intensive care unit, *LOS* length of stay, *APACHE* Acute Physiology and Chronic Health Evaluation, *SOFA* Sequential Organ Failure Assessment, *6MWT* 6-min walk test

^a^β-Coefficients (p value) modeled as univariate regression analyses. 6MWT and muscle strength modeled as percent predicted. β-Coefficients represent the percent change in the physical functioning variable per unit change in exposure variable

^b^Each domain of the EuroQol-5D questionnaire modeled as 0 “no problems” and 1 “any problems”

^c^Visual analog scale of the EQ-5D scores ranges from 0 to 10 based on patients perspective of current overall health

^d^The physical function domain is standardized to a 0–100 scale, and higher scores indicate better health-related quality of life

^e^Physical and mental composite scores of the short-form 36 survey, standardized to Canadian norms. Scores can range from 0 to 100 and are standardized to 50, which represents the average Canadian’s health-related quality of life

^f^Hospital Anxiety and Depression Scale (HADS), scores range from 0 to 21, significant symptoms of anxiety/depression defined as scores ≥8

The physical function domain of the SF-36 was associated with the percent-predicted 6MWT distance (p < 0.001), grip strength (p = 0.019) and hamstring strength (p = 0.026). The 6MWT percent-predicted distance was associated with the physical composite score of the SF-36 (p = 0.003) and the anxiety score of the Hospital and Anxiety Depression Scale. Patient’s percent-predicted 6MWT and muscle strength for all muscle groups, except triceps, were statistically associated with reported values in the general health domain (patient’s perspective of overall health) of the SF-36. Additionally, patients predicted grip strength and 6MWT were associated with the vitality, social functioning and role emotional domains, with the 6MWT also associated with the physical role and emotional role domains (data not shown).

Univariate regression analysis showed that ICU LOS (p = 0.002), number of days mechanically ventilated (p = 0.002), the presence of sepsis (p = 0.044) and corticosteroid use in the ICU (p = 0.019) were independently associated with patient’s percent-predicted 6MWT distance (Table 4). Sepsis was the only ICU risk factor associated with a reduction in the 6MWT by an estimated 17 % and reductions in strength of all muscle groups (except quadriceps). Muscle strength did not correlate with patient’s ICU or hospital LOS. Both the 6MWT and peripheral muscle strength did not show an association with the Functional Comorbidity Index, APACHE II or SOFA scores. There were no associations found between neuromuscular medication use or the presence of ARDS in the ICU and the 6MWT, peripheral muscle strength or self-reported physical functioning (SF-36, EQ-5D) (data not shown).

**Table 4:** Summary of predictors of muscle strength and physical functioning at 3 months after hospital discharge

Variable n = 50^a^	6-Minute walk test	Grip	Triceps	Biceps	Hamstrings	Quadriceps	Ankle dorsiflexors
ICU LOS, days	−0.003 (0.041)	−0.002 (0.85)	−0.010 (0.305)	−0.005 (0.537)	−0.001 (0.806)	0.000 (0.922)	−0.001 (0.685)
Hospital LOS, days	−0.003 (0.079)	−0.001 (0.965)	0.002 (0.727)	0.003 (0.498)	0.000 (0.969)	0.003 (0.274)	0.001 (0.755)
Mechanical ventilation, days	−0.004 (0.045)	−0.001 (0.905)	−0.009 (0.378)	−0.004 (0.610)	−0.001 (0.899)	0.001 (0.809)	−0.001 (0.742)
Presence of sepsis	−0.168 (0.029)	−0.389 (0.009)	−0.328 (0.045)	−0.315 (0.033)	−0.328 (0.016)	−0.134 (0.262)	−0.197 (0.049)
APACHE II score	−0.003 (0.642)	−0.002 (0.868)	−0.008 (0.515)	0.003 (0.802)	0.003 (0.755)	0.011 (0.212)	0.006 (0.397)
SOFA score	0.011 (0.257)	0.022 (0.229)	0.021 (0.325)	0.027 (0.167)	0.020 (0.219)	0.019 (0.174)	0.017 (0.154)
Functional Comorbidity Index	−0.019 (0.593)	−0.070 (0.247)	−0.111 (0.114)	−0.050 (0.437)	−0.000 (0.998)	0.013 (0.803)	−0.044 (0.274)
Any corticosteroid use^b^	−0.215 (0.019)	−0.304 (0.092)	−0.382 (0.062)	−0.359 (0.052)	−0.320 (0.047)	−0.222 (0.137)	−0.221 (0.058)

*ICU* intensive care unit, *LOS* length of stay, *APACHE* Acute Physiology and Chronic Health Evaluation, *SOFA* Sequential Organ Failure Assessment, *6MWT* 6-min walk test

^a^β-Coefficients (p value) modeled as univariate regression analyses. 6MWT and muscle strength modeled as percent predicted. β-Coefficients represent the percent change in the physical functioning variable per unit change in exposure variable

^b^Also analyzed using cumulative ICU and average daily corticosteroid dose with no significance found

The multivariate linear regression analysis assessing predictors of 6MWT distance included the ICU risk factors that were statistically significant in the univariate regression, with the exception of mechanical ventilation duration as it is highly correlated with ICU LOS. The presence of sepsis (β = −0.159, p = 0.030) and any corticosteroid use (β = −0.188, p = 0.037) in the ICU were associated with patient’s age- and sex-matched 6MWT distance (R^2^ = 0.24), and ICU LOS (β = −0.002, p = 0.233) was no longer statistically significant. Additionally, total cumulative steroid dose and average daily corticosteroid dose were analyzed in regression model and there was no association with physical function or muscle strength (data not shown).

## Discussion

This is the first study to report the use of hand-held dynamometry assessment of multiple muscle groups in the general ICU survivor population. We demonstrated that approximately 3 months after hospital discharge the majority of ICU survivors experienced persistent muscle weakness across all muscle groups. The quadriceps were the weakest muscle group measured, with the median percent-predicted strength achieved at 59 %. Fan et al. [23] described grip strength in ARDS survivors, in keeping with our results, reporting a range of 50–70 % percent-predicted strength 3–12 months after ICU discharge. Global muscle weakness in ARDS survivors, measured using MMT, has a reported prevalence between 8 and 22 % during the first year after discharge [23, 24]. However, we found that over 50 % of patients did not achieve 80 % of their age- and sex-matched predicted hand-held dynamometry-measured muscle strength. These differences may be due to the limited sensitivity of MMT to detect less severe weakness [24], or that our study included all ICU patients versus only ARDS patients.

A reduction in percent-predicted 6MWT distance and grip strength was associated with a decrease in the physical functioning domain on the SF-36 survey, similar to results found in prior studies [22–24]. We found that poor 6MWT performance was also associated with reduced HRQL in nearly all of the domains of the EQ-5D and SF-36, including the physical composite score, suggesting there is a strong link between patients overall physical functioning and HRQL. Further supporting this finding, the muscle strength of patient’s was independently associated with the patient’s perception of their mobility and general health on the SF-36. Poor 6MWT performance was associated with increased symptoms of anxiety, showing the connection between physical functioning and mental health [6]. It is important to note there was an expected association between muscle strength and the 6MWT; however, the 6MWT was associated with more domains of HRQL than muscle strength alone. This finding highlights that the 6MWT accounts for more than just physical functioning or muscle strength, and is a composite assessment that may also be influenced by cardiopulmonary status, mental health, motivation, neurological status and bodily pain [24, 44, 45].

ICU LOS is thought to be a surrogate marker for immobility [23]. This is a well-described risk factor for muscle wasting and weakness in both healthy controls and patients in ICU [10, 46]. However, in our multivariate regression analysis ICU LOS was not found to be a predictor of muscle strength or 6MWT. The model included both corticosteroid use and the presence of sepsis, suggesting that ICU LOS, an indicator of immobility, may be confounded or collinearly correlated by these variables. Therefore, interventions to reduce immobility in the ICU may not decrease the prevalence of long-term impaired muscle strength or physical functioning. However, the benefits of interventions such as early mobilization still need to be examined in long-term randomized controlled trials using the appropriate physical outcome measures [47–50].

The presence of sepsis during critical illness was significantly associated with both impaired physical functioning and muscle weakness in nearly all muscle groups (the most of any ICU risk factor) as part of the univariate and multivariate linear regression analysis. Systemic inflammation and cytokine release have been shown to induce muscle injury and increase the incidence of ICUAW detected in the ICU [7, 51–53]. Our findings support a premise that inflammation during critical illness can result in long-term detectable impairment of muscle function [3]. Our study suggests sepsis, as a mechanism possibly related to the acute systemic inflammatory process, may be more important than ICU LOS or immobility in long-term physical impairment.

Our analysis showed that any exposure to corticosteroid use during critical illness was associated with reduced physical functioning. This supports the findings of prior studies in ARDS survivors and recommendations to avoid corticosteroid use in the ICU in order to lessen the degree of patient’s long-term functional disability [5, 10, 24]. We did not see any effect on physical functioning when analyzing average daily corticosteroid dose or cumulative dose, similar to other studies [10]. Prior studies examining the effects of corticosteroids were limited to patients who had ARDS, but surprisingly none of our patients who had ARDS (23 %) received any corticosteroids. This highlights the variability of clinical practice across institutions and the importance of studying long-term physical functioning outcomes across the general ICU population.

The limitations of this study include that it is a single-center study with a relatively small sample size. Additionally, patient recruitment was limited due to the small capacity of the follow-up clinic. Patients who may have met inclusion criteria were not screened, potentially creating selection bias. However, patient selection was random and we had a relatively few number of patients decline enrollment or who were lost to follow-up.

## Conclusion

We found that in survivors of critical illness approximately 3 months after hospital discharge patients had significant impairment in muscle strength and physical functioning measured using hand-held dynamometry and the 6MWT. Patients with impaired physical functioning and muscle weakness were found to have reduced HRQL. Sepsis and corticosteroid use were found to be an important risk factor for reduced long-term physical function, whereas ICU length of stay (a surrogate for immobility) was not.

## Abbreviations

ARDS: acute respiratory distress syndrome
APACHE II: Acute Physiology and Chronic Health Evaluation II
ESS: Epworth Sleepiness Scale
EQ-5D: EuroQol-5D
HADS: Hospital Anxiety and Depression Scale
ICU: intensive care unit
ICUAW: intensive care unit-acquired weakness
IQR: interquartile range
LOS: length of stay
MCS: mental composite summary
MMT: manual muscle testing
PCS: physical composite summary
PQSI: Pittsburgh Sleep Quality Index
PSG: polysomnography
SF-36: short-form 36
SOFA: Sequential Organ Failure Assessment
6MWT: 6-min walk test
